# Describing Transitions in Adherence to Physical Activity Self-monitoring and Goal Attainment in an Online Behavioral Weight Loss Program: Secondary Analysis of a Randomized Controlled Trial

**DOI:** 10.2196/30673

**Published:** 2022-01-28

**Authors:** Melissa L Stansbury, Jean Harvey, Rebecca A Krukowski, Christine A Pellegrini, Xuewen Wang, Delia Smith West

**Affiliations:** 1 Department of Exercise Science Arnold School of Public Health University of South Carolina Columbia, SC United States; 2 Department of Nutrition and Food Sciences College of Agriculture and Life Sciences University of Vermont Burlington, VT United States; 3 Department of Public Health Sciences School of Medicine University of Virginia Charlottesville, VA United States

**Keywords:** physical activity, adherence, self-monitoring, goal attainment, lifestyle intervention

## Abstract

**Background:**

Standard behavioral weight loss interventions often set uniform physical activity (PA) goals and promote PA self-monitoring; however, adherence remains a challenge, and recommendations may not accommodate all individuals. Identifying patterns of PA goal attainment and self-monitoring behavior will offer a deeper understanding of how individuals adhere to different types of commonly prescribed PA recommendations (ie, minutes of moderate-to-vigorous physical activity [MVPA] and daily steps) and guide future recommendations for improved intervention effectiveness.

**Objective:**

This study examined weekly patterns of adherence to step-based and minute-based PA goals and self-monitoring behavior during a 6-month online behavioral weight loss intervention.

**Methods:**

Participants were prescribed weekly PA goals for steps (7000-10,000 steps/day) and minutes of MVPA (50-200 minutes/week) as part of a lifestyle program. Goals gradually increased during the initial 2 months, followed by 4 months of fixed goals. PA was self-reported daily on the study website. For each week, participants were categorized as adherent if they self-monitored their PA and met the program PA goal, suboptimally adherent if they self-monitored but did not meet the program goal, or nonadherent if they did not self-monitor. The probability of transitioning into a less adherent status was examined using multinomial logistic regression.

**Results:**

Participants (N=212) were predominantly middle-aged females with obesity, and 67 (31.6%) self-identified as a racial/ethnic minority. Initially, 73 (34.4%) participants were categorized as adherent to step-based goals, with 110 [51.9%] suboptimally adherent and 29 [13.7%] nonadherent, and there was a high probability of either remaining suboptimally adherent from week to week or transitioning to a nonadherent status. However, 149 (70.3%) participants started out adherent to minute-based goals (34 [16%] suboptimally adherent and 29 [13.7%] nonadherent), with suboptimally adherent seen as the most variable status. During the graded goal phase, participants were more likely to transition to a less adherent status for minute-based goals (odds ratio [OR] 1.39, 95% CI 1.31-1.48) compared to step-based goals (OR 1.24, 95% CI 1.17-1.30); however, no differences were seen during the fixed goal phase (minute-based goals: OR 1.06, 95% CI 1.05-1.08; step-based goals: OR 1.07, 95% CI 1.05-1.08).

**Conclusions:**

States of vulnerability to poor PA adherence can emerge rapidly and early in obesity treatment. There is a window of opportunity within the initial 2 months to bring more people toward adherent behavior, especially those who fail to meet the prescribed goals but engage in self-monitoring. Although this study describes the probability of adhering to step- and minute-based targets, it will be prudent to determine how individual characteristics and contextual states relate to these behavioral patterns, which can inform how best to adapt interventions.

**Trial Registration:**

ClinicalTrials.gov NCT02688621; https://clinicaltrials.gov/ct2/show/NCT02688621

## Introduction

Adherence to physical activity (PA) is a pervasive challenge in behavioral weight control programs. Many individuals fail to achieve program recommendations and adopt a physically active lifestyle [1,2]. As a result, these individuals do not obtain the multitude of immediate and long-term health benefits associated with engaging in PA, including chronic disease prevention and weight management [3,4]. A deeper understanding of the underlying pattern of an individual’s adherence to program recommendations may reveal information about what contributes to deterioration in PA adherence and indicate important targets for intervention.

Ordinarily, all individuals receive the same programmatic goals, which gradually progress to targeted PA levels [5-7]. Recommendations are most often provided for moderate-to-vigorous intensity physical activity (MVPA) in terms of minute-based goals, such as 150 minutes/week of MVPA [8]. More recently, step-based goals (eg, 10,000 steps/day) have also been incorporated as a means to bolster the accumulation of activity throughout the day. Individuals who achieve program MVPA goals during treatment tend to lose more weight and are more likely to maintain high levels of activity in the future [1,9-12]. However, the stability in adherence to step- and minute-based goals over time, as well as differences in adherence between these 2 types of goal prescriptions, have yet to be identified. Responsiveness to program recommendations, along with a more precise description of how the type and timing of PA goals impact adherence, can uncover key areas for consideration when prescribing PA for behavioral weight control and guide intervention tailoring for those at risk of poor adherence.

Self-monitoring, a key element of lifestyle programs intended to promote behavioral awareness and fulfillment of recommendations [4,13], serves as a reliable indicator of weight loss and PA engagement [10,14]. Failure to initiate self-monitoring of MVPA and inconsistent recording of MVPA are associated with weight gain and lower activity levels during a behavioral weight loss program [15]. Whether self-monitoring of daily steps follows a similar pattern to MVPA remains to be determined. Describing how individuals adhere to self-monitoring of different types of program PA goals from one week to the next may point toward which type of metric (steps or minutes of MVPA) individuals are more likely to engage in during a behavioral weight control program.

Others have examined transitions in levels of adherence to dietary self-monitoring and found that individuals who partially self-monitor in 1 week have a greater chance of improving to full self-monitoring during the first 2 months of treatment, whereas after the first 2 months, partial self-monitoring in 1 week foreshadows discontinuation of self-monitoring [16]. Using a similar approach to examining PA will indicate whether there are meaningful patterns of PA self-monitoring and engagement, as well as when these patterns emerge. In turn, potential targets for adaptive interventions may be identified, which could ultimately improve adherence in behavioral weight control programs and enhance outcomes for both PA promotion and weight management.

Examination of weekly adherence to the different types of PA goals offers insight into goal operationalization, progression, and timing, since lifestyle programs tend to prescribe goals on a weekly basis. Therefore, this study aims to describe weekly patterns of adherence to (1) step-based goals and self-monitoring steps and (2) minute-based goals and self-monitoring minutes of MVPA, as well as examine whether weekly transitional patterns differ between step-based goals versus minute-based goals, across 6 months of an online, group-based behavioral weight control program.

## Methods

### Study Design

This study was a secondary analysis of a randomized controlled trial (RCT) that evaluated the impact of financial incentives on weight loss in a group-based behavioral weight control program delivered online [17]. Briefly, participants received either (1) the online program augmented with financial incentives for achieving behavioral and weight loss goals or (2) the online program alone (ie, control group). Eligible participants had a body mass index (BMI) of 25-50 kg/m^2^; were at least 18 years of age with access to a smartphone, computer, and the internet; had no medical contraindications to moderate-intensity PA (eg, brisk walking); and demonstrated the capacity to monitor their PA using their smartphone or personal fitness tracker prior to randomization. The study was approved by the institutional review boards at both clinical sites, and written informed consent was obtained from all participants. The investigation included only the control group (N=212) to avoid the potential of financial incentives obscuring PA adherence patterns (see Multimedia Appendix 1).

### Program Overview

All participants received a goal-oriented behavioral weight control program delivered online, which has been shown effective in previous studies [18,19]. Weekly online group sessions occurred synchronously via text-based chat led by an experienced facilitator for 6 months. Session topics, such as goal setting, problem solving, action planning, and relapse prevention, were supplemented with online materials, activities, and other resources on the study website to reinforce key strategies for self-regulation. A program goal of 10% weight loss was recommended through calorie reduction and increased PA. Participants were provided a dietary goal of 1200-1800 calories/day (≤25% from fat) based on their initial body weight. The same step- and minute-based PA goals were prescribed concurrently to all participants from weeks 3 to 24 in 2 phases. The graded goal phase began at week 3, with targets for 7000 steps/day and 50 minutes/week of MVPA; both goals progressed incrementally, reaching 10,000 steps/day and 150 minutes/week of MVPA at week 8. The fixed goal phase consisted of weeks 9-24, when both program PA goals were held constant at 10,000 steps/day and 200 minutes/week of MVPA. The MVPA goal was chosen to be consistent with the Centers for Disease Control and Prevention and American College of Sports Medicine guidelines [8]. Although there is much debate about the number of recommended steps, the 10,000-step goal is commonly used [20].

Participants were instructed to use their personal fitness tracker or a smartphone app to track their total steps and minutes of planned exercise of at least a moderate-to-vigorous intensity and then record both their total steps and minutes of MVPA on the study website daily. In other words, participants were asked to report both metrics for each day of the study period, as opposed to reporting only 1 metric, even if they did not perform any PA on that day. Self-monitoring records were reviewed by the facilitator, who provided tailored feedback via email on a weekly basis with positive reinforcement of healthy lifestyle choices, constructive guidance promoting potential areas for change, and reminders of the program recommendations.

### Measures

#### Physical Activity

The total number of steps and minutes of MVPA both were self-reported by participants daily on the study website. Weekly totals for steps and for minutes of MVPA were calculated by summing the daily reported values, with each week beginning on the day of the scheduled group session.

#### Adherence to PA Goals

Adherence to the program step goals (yes/no) and the MVPA minute goal (yes/no) was separately determined for each participant and for each week of the program based on the proportion of the program goal achieved. For example, a participant could be categorized as nonadherent to the step goal and adherent to the MVPA goal. For steps, the average daily step count for a given week was divided by that week’s program step goal and multiplied by 100%. For minutes of MVPA, the total minutes reported in a given week were divided by the program MVPA goal and multiplied by 100%. Proportions of ≥100% indicated that the corresponding goal was met (yes), and proportions of <100% indicated that the goal was unmet (no). A value of 0 was assumed for missing and implausible entries, including daily step counts of <1000 or >30,000 [21,22] and >1080 MVPA minutes/day [23,24].

#### Adherence to PA Self-Monitoring

Adherence to self-monitoring of steps (yes/no) and minutes of MVPA (yes/no) was based on whether an entry was submitted and calculated separately for each type of PA goal (ie, a participant could be categorized as adherent to 1 of the goals and not adherent to the other). For steps, a participant was considered to have self-monitored (yes) in a given week if ≥1 record of any step count was submitted, including a value of 0. Likewise, a participant was considered to have self-monitored minutes of MVPA (yes) for a given week if ≥1 record of any number of minutes, including 0, was submitted. If no entry was submitted for the corresponding PA goal on any of the 7 days, the participant was considered nonadherent to self-monitoring (no) for that week. The approach described here is consistent with previous work, which categorized a participant as adherent if ≥1 record of any value of exercise minutes was submitted for a given week and nonadherent if no records were submitted [25].

#### Categorization of PA Adherence

The variables *adherence to PA goal* and *adherence to PA self-monitoring* were used to categorize participants into 3 mutually exclusive categories each week for step-based goals and separately for minute-based goals. Participants were categorized as (1) *adherent* if they met the goal and self-monitored, (2) *suboptimally adherent* if they did not meet the goal but did self-monitor, and (3) *nonadherent* if they did not self-monitor (Table 1). It was assumed that those who did not self-monitor also did not meet the PA goal for that week. To the best of our knowledge, thresholds for adherence to PA goals and self-monitoring during behavioral weight control have not been standardized. Therefore, the categories were selected to mirror the established PA classifications outlined in the national guidelines (ie, *active/highly active*, *insufficiently active*, and *inactive*) [26], which are related to the degree of health benefits obtained [8]. Categorization of PA adherence to step-based goals did not influence how a participant was categorized for minute-based goals, and vice versa.

**Table 1:** Categorization of PA^a^ adherence based on weekly program PA goals and PA self-monitoring.

Week			Adherent		Suboptimally adherent	Nonadherent
**Steps**						
	3	≥7000 steps (≥100%) + ≥1 day of self-monitoring		1-6999 steps (0%<x<100%) + ≥1 day of self-monitoring		No self-monitoring
	4-5	≥8000 steps (≥100%) + ≥1 day of self-monitoring		1-7999 steps (0%<x<100%) + ≥1 day of self-monitoring		No self-monitoring
	6-7	≥9000 steps (≥100%) + ≥1 day of self-monitoring		1-8999 steps (0%<x<100%) + ≥1 day of self-monitoring		No self-monitoring
	8-24	≥10,000 steps (≥100%) + ≥1 day of self-monitoring		1-9999 steps (0%<x<100%) + ≥1 day of self-monitoring		No self-monitoring
**Minutes of MVPA^b^**						
	3-4	≥50 minutes (≥100%) + ≥1 day of self-monitoring		1-49 minutes (0%<x<100%) + ≥1 day of self-monitoring		No self-monitoring
	5-6	≥100 minutes (≥100%) + ≥1 day of self-monitoring		1-99 minutes (0%<x<100%) + ≥1 day of self-monitoring		No self-monitoring
	7-8	≥150 minutes (≥100%) + ≥1 day of self-monitoring		1-149 minutes (0%<x<100%) + ≥1 day of self-monitoring		No self-monitoring
	9-24	≥200 minutes (≥100%) + ≥1 day of self-monitoring		1-199 minutes (0%<x<100%) + ≥1 day of self-monitoring		No self-monitoring

^a^PA: physical activity.

^b^MVPA: moderate-to-vigorous physical activity.

### Statistical Analysis

Descriptive analyses were conducted to characterize the sample and determine frequencies for the number of participants meeting PA goals and self-monitoring their PA each week. Marginal proportions of weekly PA adherence categories were separately tabulated for each type of PA recommendation (steps and minutes of MVPA) beginning at week 3 and continuing through the end of the 6-month period. The weekly transition probabilities of adherence status were then independently summarized for step- and minute-based goals using separate models for the graded PA goal phase (weeks 3-8), fixed PA goal phase (weeks 9-24), and across all time points (weeks 3-24). Models were conditioned on the probability of maintaining the same adherence status from the preceding week or transitioning to another status within each type of goal prescription.

The likelihood of having an adherent, suboptimally adherent, or nonadherent status over time was examined for step- and minute-based goals, separately, using multinomial logistic regression for correlated data. The odds of having a less adherent status were modeled for each type of PA recommendation during the graded PA goal phase, fixed PA goal phase, and across all time points. Comparisons were then made between models for step- versus minute-based goals on the odds of transitioning into a less adherent status. All analyses were performed using SAS 9.4 (Cary, NC, USA), and the level of statistical significance was set at *P*<.05.

## Results

### Participant Characteristics

All participants randomized to the control group (N=212) in the larger RCT [17] were included in this study (Table 2). On average, participants in the current analyses were 47.9 years old, with a BMI of 35.8 kg/m^2^. The majority (n=194, 91.5%) were female, and approximately one-third (n=67, 31.6%) self-identified as a racial/ethnic minority. Most participants had at least a college degree (n=169, 79.7%) and were employed full-time (n=152, 71.7%). Retention was 81.1% (n=172) at the 6-month assessment visit.

**Table 2:** Baseline sociodemographic characteristics of study participants (N=212).

Characteristic			Value
Age (years), mean (SD)			47.9 (11.1)
BMI^a^ (kg/m^2^), mean (SD)			35.8 (5.9)
**Gender, n (%)**			
	Female	194 (91.5)	
	Male	18 (8.5)	
**Race/ethnicity, n (%)**			
	White	145 (68.4)	
	Minority^b^	67 (31.6)	
**Education, n (%)**			
	College degree or higher	169 (79.7)	
	Some college or less	43 (20.3)	
**Marital status, n (%)**			
	Married or cohabiting	118 (55.7)	
	Separated, divorced, widowed, or never married	94 (44.3)	
**Employment status, n (%)**			
	Full time	152 (71.7)	
	Part time or unemployed	60 (28.3)	
**Geographic region, n (%)**			
	Northeast (Vermont)	106 (50.0)	
	Southeast (South Carolina)	106 (50.0)	

^a^BMI: body mass index.

^b^Minority groups include African American, Asian, Hispanic, and Pacific Islander.

### Rates of Adherence to Physical Activity

#### Step-Based Goals

**Figure 1:**
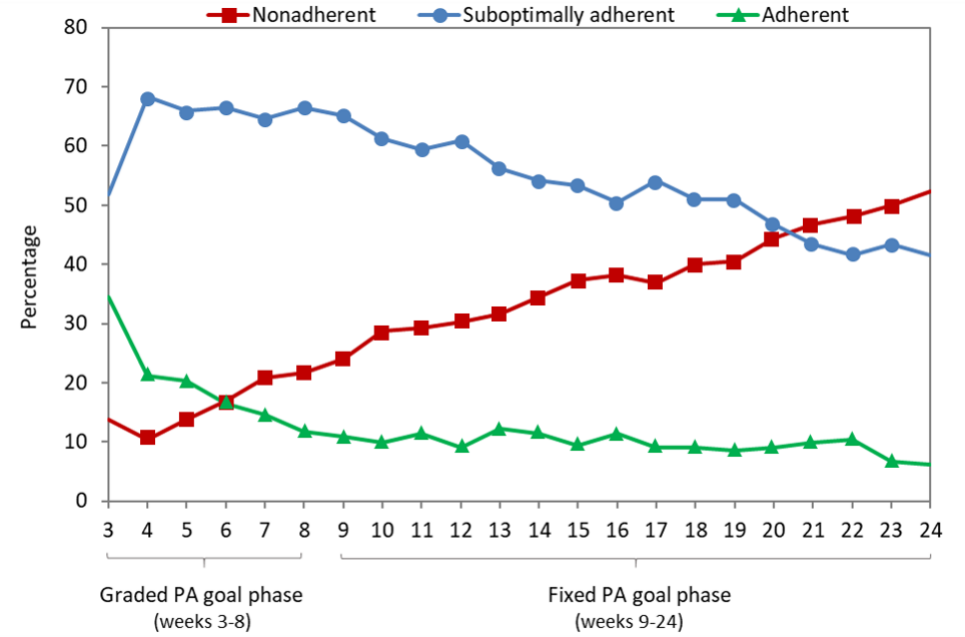
Weekly marginal proportions of participants in each PA adherence category for step-based goals (N=212). PA: physical activity.

On average, of the 212 participants, 144 (67.7%) self-monitored their steps on ≥1 day/week during the 6-month period and 27 (12.4%) met the prescribed steps goal each week. Approximately one-third (n=73, 34.4%) of the participants started out adherent to the 7000-daily-step goal in week 3 (Figure 1). Rates declined as the goal progressed to 10,000 daily steps, and 13 (6.1%) participants remained adherent by 6 months. More than half of the participants (n=110, 51.9%) initially self-monitored their steps but did not meet the prescribed step goal (suboptimally adherent). The same pattern observed for adherent participants was also seen for suboptimally adherent participants, in which the proportion of suboptimally adherent participants declined over time to 88 (41.5%) participants. In contrast, the proportion of participants who were initially nonadherent (n=29, 13.7%) steadily increased until 111 (52.4%) participants were no longer self-monitoring steps at the conclusion of the 6-month study period.

#### Minute-Based Goals

**Figure 2:**
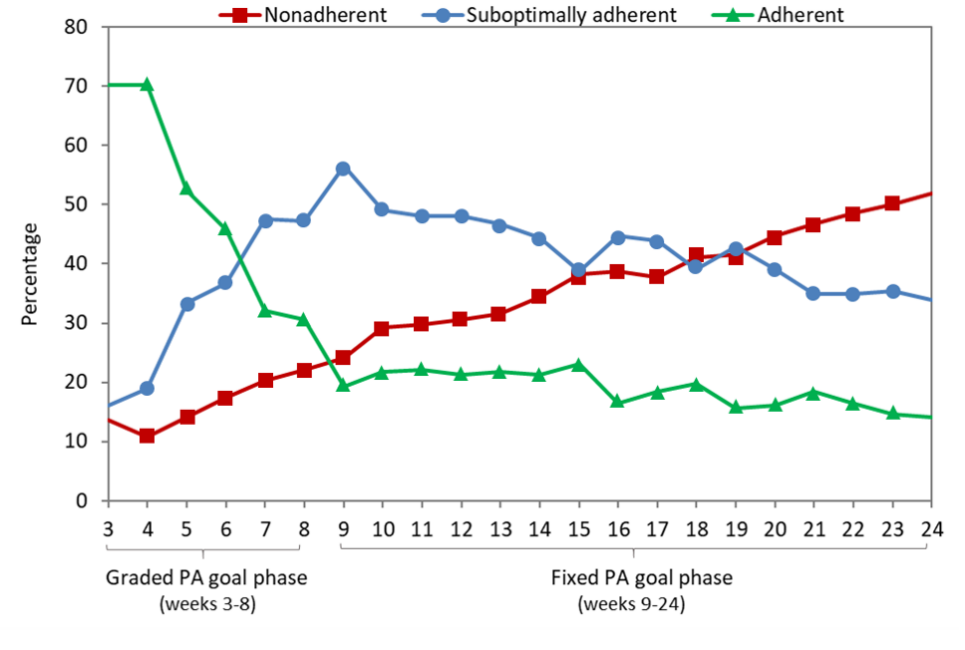
Weekly marginal proportions of participants in each PA adherence category for minute-based goals (N=212). PA: physical activity.

Comparable to daily steps, 143 (67.4%) participants self-monitored their total minutes of MVPA on ≥1 day per week over the 6-month period (Figure 2); however, more than twice as many participants (n=58, 27.4%) met the weekly MVPA minute goals relative to the step goals. Most participants (n=149, 70.3%) were adherent to the initial 50-minute MVPA goal. Adherent behavior rapidly declined as MVPA goals progressed through the graded phase, followed by a slower rate of deterioration in the fixed goal phase to 30 (14.2%) participants by 6 months. The proportion of those classified as suboptimally adherent increased sharply during the graded phase before gradually declining throughout the remaining weeks. Finally, the proportion of participants considered nonadherent to MVPA minute goals closely mirrored that of participants considered nonadherent to step goals over time.

### Transitions in Adherence to Physical Activity

The weekly transitional probabilities of adherence to prescribed PA goals and PA self-monitoring across successive weeks of the behavioral weight control program are summarized for steps and minutes of MVPA in Table 3 (see Multimedia Appendix 2 for flow diagrams of transitions). The most stable status by phase and across the full 6-month period for both step- and minute-based goals was the nonadherent status. If a participant was nonadherent to the initial program goals at week 3, they were estimated to remain nonadherent at the end of the 8-week graded phase 90% of the time and transition to suboptimally adherent 10% of the time. The likelihood of remaining nonadherent increased in the fixed goal phase to 98%. A participant had a 100% probability of being nonadherent at week 24, given that they had been nonadherent to the initial PA goals at week 3. It was improbable that a participant would transition directly from a nonadherent status in 1 week to an adherent status the following week.

**Table 3:** Summary of weekly transitional probabilities of PA^a^ adherence by program phase^b^.

Adherence status		Nonadherent (time_t_)		Suboptimally adherent (time_t_)		Adherent (time_t_)	
		Steps	MVPA^c^	Steps	MVPA	Steps	MVPA
**Graded PA goal phase^d^ (time_t–1_), %**							
	Nonadherent	*89.7* ^e^	*89.7*	10.3	10.3	0	0
	Suboptimally adherent	15.5	14.7	*80.9*	*70.6*	3.6	14.7
	Adherent	4.1	10.7	67.1	49.0	*28.8*	*40.3*
**Fixed PA goal phase^f^ (time_t–1_), %**							
	Nonadherent	*97.8*	*97.9*	2.2	0	0	2.1
	Suboptimally adherent	44.0	46.0	*52.5*	*48.0*	3.5	6.0
	Adherent	16.0	27.7	52.0	36.9	*32.0*	*35.4*
**Full 6-month period^g^ (time_t–1_), %**							
	Nonadherent	*100*	*100*	0	0	0	0
	Suboptimally adherent	53.6	64.7	*44.5*	*29.4*	1.8	5.9
	Adherent	31.5	39.6	53.4	41.6	*15.1*	*18.8*

^a^PA: physical activity.

^b^Transition probabilities describe the likelihood of remaining in an adherence status in 1 week (time_t_), given the adherence status in the previous week (time_t–1_) or transitioning to another status.

^c^MVPA: moderate-to-vigorous physical activity.

^d^Probabilities indicate adherence status at the end of the graded PA goal phase (week 8), given the adherence status for the initial PA goal at week 3.

^e^Values in italics indicate remaining in the same adherence status.

^f^Probabilities indicate adherence status at the end of the fixed PA goal phase (week 24), given the adherence status at the end of the graded PA goal phase (week 8).

^g^Probabilities indicate adherence status at the end of the study period (week 24), given the adherence status for the initial PA goal at week 3.

#### Step-Based Goals

During the initial 8 weeks, as the goals progressed from 7000 to 10,000 daily steps, the probability that a participant would remain adherent was 29%, while the probability of transitioning from adherent to suboptimally adherent was 67% (Table 3; also see Multimedia Appendix 2A for step-based goals). For example, if a participant was suboptimally adherent to the 7000-step goal at week 3, they had an 81% chance of remaining suboptimally adherent and a 16% chance of transitioning to a nonadherent status when the goal increased to 10,000 steps at week 8. Across the fixed goal phase, those who were suboptimally adherent at the end of the graded phase (week 8) had a 53% chance of continuing to be suboptimally adherent, a 44% chance of transitioning to a nonadherent status, and a 4% chance of transitioning to an adherent status at the conclusion of 6 months. A participant was expected to remain adherent at the end of the program 15% of the time if they were adherent in week 3; however, the chances of being adherent at the end of the program increased to 32% if they were adherent at week 8. When looking at weekly transitions during the phase with graded PA goals, participants had the highest likelihood of remaining suboptimally adherent or transitioning from being nonadherent to suboptimally adherent between weeks 3 and 4 (95% and 31%, respectively), as shown in Table 4 (see Multimedia Appendix 3A for step-based goals).

**Table 4:** Weekly transitional probabilities of PA^a^ adherence during the graded goal phase^b^.

Adherence status		Nonadherent (time_t_)		Suboptimally adherent (time_t_)		Adherent (time_t_)	
		Steps	MVPA^c^	Steps	MVPA	Steps	MVPA
**Week 4 (time_t–1_), %**							
	Nonadherent	*69.0* ^d^	*72.4*	31.0	24.1	0	3.4
	Suboptimally adherent	1.8	29.0	*94.5*	*50.0*	3.6	47.1
	Adherent	0	0.7	43.8	10.7	*56.2*	*88.6*
**Week 5 (time_t–1_), %**							
	Nonadherent	*81.8*	*82.6*	18.2	17.4	0	0
	Suboptimally adherent	7.6	22.5	*82.1*	*62.5*	10.3	15.0
	Adherent	0	1.3	37.8	28.2	*62.2*	*70.5*
**Week 6 (time_t–1_), %**							
	Nonadherent	*93.1*	*93.3*	6.9	6.7	0	0
	Suboptimally adherent	5.7	9.9	*86.4*	*69.0*	7.9	21.1
	Adherent	2.3	1.8	41.9	24.3	*55.8*	*73.9*
**Week 7 (time_t–1_), %**							
	Nonadherent	*86.1*	*83.8*	13.9	13.5	0	2.7
	Suboptimally adherent	9.2	14.1	*82.3*	*78.2*	8.5	7.7
	Adherent	0	1.0	45.7	36.1	*54.3*	*62.9*
**Week 8 (time_t–1_), %**							
	Nonadherent	*81.8*	*81.4*	18.2	18.6	0	0
	Suboptimally adherent	7.3	10.9	*85.4*	*71.3*	7.3	17.8
	Adherent	0	1.5	51.6	29.4	*48.4*	*69.1*

^a^PA: physical activity.

^b^Transition probabilities describe the likelihood of remaining in an adherence status in 1 week (time_t_), given the adherence status in the previous week (time_t–1_) or transitioning to another status.

^c^MVPA: moderate-to-vigorous physical activity.

^d^Values in italics indicate remaining in the same adherence status.

#### Minute-Based Goals

In the graded goal phase, participants had a 40% chance of remaining adherent to the 150-minute recommendation at week 8 if they were initially adherent to the 50-minute target, in addition to a 49% chance of transitioning from being adherent to suboptimally adherent (Table 3; also see Multimedia Appendix 2B for minute-based goals). Participants were likely to remain suboptimally adherent at week 8 approximately 71% of the time if their initial status had been suboptimally adherent, with an equal chance of moving from a suboptimally adherent to a nonadherent or adherent status. During the period when the prescribed MVPA was fixed at 200 minutes per week (weeks 9-24), a participant was most likely to remain suboptimally adherent (48%) or transition from being suboptimally adherent to nonadherent (46%), with a 6% chance of transitioning to an adherent status. A participant was expected to remain adherent at the end of the program 19% of the time, given that they were adherent to the initial 50-minute MVPA goal, and doubled their chances of being adherent at the end of the program if they were adherent at the 8-week mark. When looking at weekly transitions during the phase with graded PA goals, adherence status from weeks 3 to 4 demonstrated the greatest chances of remaining adherent (89%), improving from suboptimally adherent to adherent (47%), and improving from nonadherent to suboptimally adherent (24%). See Table 4; in addition, see Multimedia Appendix 3B for minute-based goals.

### Comparison of Adherence Categories for Step-Based Versus Minute-Based Goals

Analyses examining adherence status over time indicated that participants were significantly more likely to be in a less adherent category relative to adherent as the program progressed (Table 5). Thus, participants were most likely to transition to a lower adherence category each week in both goal phases and across the 6-month period for step-based goals (all *P*<.001), as well as for minute-based goals (all *P*<.001).

**Table 5:** Comparison of step- versus minute-based goals on the odds of being in a less adherent weekly status.

Phase	Step-based goals	Minute-based goals	Steps vs minutes
	OR^a^ (95% CI)	OR (95% CI)	*P* value^b^
Graded PA^c^ goal phase^d^	*1.24 (1.17-1.30)* ^e^	*1.39 (1.31-1.48)*	.004
Fixed PA goal phase^f^	*1.07 (1.05-1.08)*	*1.06 (1.05-1.08)*	.65
Full 6-month period^g^	*1.09 (1.09-1.09)*	*1.11 (1.11-1.11)*	.07

^a^OR: odds ratio (odds of being in a less adherent category over time; “adherent” is the reference group).

^b^*P* value for the odds of being in a less adherent category, relative to “adherent,” during the specified phase for step-based goals versus minute-based goals.

^c^PA: physical activity.

^d^Graded PA goal phase includes weeks 3-8.

^e^Values in italics indicate statistical significance at *P*<.001 during the specified phase for each type of goal.

^f^Fixed PA goal phase includes weeks 9-24.

^g^Full 6-month period includes weeks 3-24.

For steps, participants were 1.24 times more likely to be less adherent, relative to being adherent, each week during the graded goal phase, 1.07 times more likely to shift to a less adherent status during the fixed goal phase, and 1.09 times more likely to be in a less adherent category each week across the 6-month period. For minutes of MVPA, participants were 1.39 and 1.06 times more likely to be in a less adherent category each week during the graded and fixed goal phases, respectively, and 1.11 times more likely to shift to a less adherent category each week over the entire 6 months.

When steps and minutes of MVPA were compared during the graded PA goal phase, participants were significantly more likely to regress to a less adherent category the following week when examining minutes of MVPA relative to considering steps (*P*=.004). In other words, participants were more likely to become less adherent from week to week with respect to minute-based goals compared to step-based goals during weeks 3-8. No significant difference was seen in the propensity to be adherent between step- and minute-based goals during the fixed goal phase (*P*=.65). Although a trend was noted between models for steps and minutes of MVPA in the odds of being in a less adherent category when examining the full 6-month period (*P*=.07), the difference was not statistically significant.

## Discussion

### Principal Findings

To the best of our knowledge, this study is the first to describe the stability of adherence to weekly self-monitoring and attainment of PA goals prescribed in terms of daily steps and weekly minutes of MVPA during an online behavioral weight control program. Few participants met the initial step-based goals and subsequently either remained in a nonadherent or a suboptimally adherent status from one week to the next or transitioned from a suboptimally adherent to a nonadherent status over time. Conversely, most participants started out adherent to the minute-based goals but demonstrated greater variability between adherence statuses from week to week than was seen for step-based goals. Thus, participants with overweight and obesity enrolled in a behavioral weight control program were unlikely to achieve even the lowest step-based goals or to catch up with the goals as the step target progressively increased but had a greater chance of meeting the minute-based goals, particularly early in treatment.

The weekly transitional probabilities identified in this study underscore the initial 2 months of a lifestyle program as a formative period that contributes to an individual’s chances of future PA adherence. Participants who met the earliest recommendations were most likely to be adherent at 6 months relative to those who started out suboptimally adherent or nonadherent. Intriguingly, the likelihood of long-term adherence increased 2-fold if a participant remained adherent through the graded goal phase. Our findings substantiate previous studies that demonstrate that the first 2 months of program initiation serve as a critical juncture associated with better long-term program adherence, weight loss, and health-related outcomes [16,27-30]. From a theoretical perspective, successful adoption of PA recommendations may be attributed to an increase in self-efficacy and mastery experiences during program initiation from frequent, intentional exposure to self-monitoring and goal attainment [31-33]. Future studies should examine whether targeting this early period with strategies to facilitate consistent self-monitoring in conjunction with attaining recommendations substantially increases a person’s chances of long-term success in achieving PA targets and in losing weight.

Few participants achieved stability in adherent behavior, which is of concern. Indeed, nonadherence was the most consistent status and was increasingly more likely over time, regardless of the type of PA goal. Some adults with overweight may prefer monitoring minute-based activity rather than step counts [34]; however, our study did not detect a difference in self-monitoring behavior between these 2 types of PA metrics. It may be that the method of submitting daily step totals and minutes of MVPA concurrently to the study website’s digital diary contributed to the matching rates of self-monitoring steps and minutes of MVPA. Nevertheless, a considerable proportion of participants failed to track their activity and were unlikely to reengage with self-monitoring despite regular reminders of program PA goals and prompts to self-monitor, which were given in weekly emailed feedback, group meetings, and online lessons. Providing wearable devices to those who fail to engage in manual self-monitoring methods may improve adherence by facilitating continuous PA tracking and minimizing the burden associated with manual self-monitoring [35,36]. However, others have demonstrated that providing a wearable activity-tracking device in an online weight control program does not promote greater PA engagement or weight loss [37].

Suboptimally adherent behavior appeared to be the most vulnerable to transitions, particularly with respect to minute-based goals. It is striking that participants had the best chances of shifting from suboptimally adherent to fully adherent behavior during the graded goal phase, yet the likelihood of becoming nonadherent outweighed becoming adherent after 2 months. A similar pattern with weekly dietary self-monitoring was previously reported where individuals who demonstrated suboptimal adherence to dietary self-monitoring had the greatest potential for transitioning to adherent behavior during the first 2 months of a lifestyle program but not after that time [16]. It is possible that a vulnerability to transitions during program initiation represents an intervention opportunity to increase the likelihood of shifting to fully adherent behavior. Supporting individuals with coaching sessions at the first sign of vulnerability might foster an increase in their MVPA or step counts [38-40]. A change in status from adherent to suboptimally adherent exemplifies 1 event that triggers implementation of a coaching session to quickly bring the individual back on a trajectory of success and avoid a decline to nonadherent behavior when it may be too late to reengage them in program recommendations.

To capitalize on the early weeks of program initiation and move more individuals toward adherent behavior, reevaluating how PA recommendations are structured for behavioral weight control is warranted. It is apparent that providing everyone with the same incremental, linear progression of PA goals does not accommodate the dynamic process of lifestyle change for each individual. From a clinical perspective, it is imperative to appreciate that the continuation of conventional treatment likely will not help vulnerable subgroups transition to adherent behavior. Instead, it may be important to provide tailored goals that adapt based on the individual’s recent PA, thus providing more realistic, attainable PA targets [38,41-43].

Although this study took the formative step of describing behavioral patterns, more work is needed to identify precisely how best to adapt treatment to capitalize on these patterns and enhance outcomes. There are likely other factors, such as cognitive, affective, and motivational components, influencing whether individuals are adherent to PA, and these should be explored to guide treatment tailoring [44-48]. For example, does exercise become unpleasant or untenable for some individuals when MVPA goals progress to 150 minutes, which contributes to them no longer adhering to recommendations? Do other individuals experience boredom with their exercise routine or a lack of motivation to pursue the goals? To what extent does a sense of self-efficacy impact attainment of step- or minute-based goals? There may also be other contextual factors influencing adherence, such as a vacation, illness/injury, or other barriers to PA, which interrupt a person’s routine. Furthermore, there may be dynamic bidirectional influences of weight loss success and PA goal attainment in the context of a weight control program; examination of these dynamic processes is warranted because success with weight loss may well be a driver of PA goal attainment, in addition to PA goal attainment driving weight loss success [1,9,11]. More research is needed before firm clinical conclusions can be made about how to adjust PA recommendations in behavioral weight control.

### Limitations

This research advances our understanding of the effects of different types of PA goals commonly prescribed in behavioral weight control interventions on goal attainment combined with self-monitoring among a large sample of adults with overweight and obesity. However, findings should be considered with the following limitations in mind. First, participants were predominantly women with obesity, and results may not generalize to other populations. Next, the influence of exercise history or the level of activity when entering the study on adherence patterns could not be determined. If program goal attainment differs between individuals who are inactive at study entry compared to those who are already engaging in some PA at baseline, recommendations may be tailored based on baseline activity levels. Although the goals offered in the program are similar to those provided by other behavioral weight control programs [5,7], patterns of goal attainment noted in this study may not generalize to other programs that recommend substantively different types or doses of PA or to PA promotion programs without an emphasis on weight loss. It is possible that the MVPA goal was more achievable than the step goal or that the participants preferred MVPA or preferred tracking MVPA. Additionally, other programs have used different MVPA goals, and it will be important to determine whether these findings generalize to other, usually higher, MVPA goals. Exploring the behavioral patterns of adherence to other PA recommendations and programs will be advantageous in determining whether there are specific thresholds for the number of steps and minutes of MVPA that optimize adherence. In addition, this study provided both PA goals, and it will be important in future research to determine if one goal or the other is more effective in inducing weight loss. Furthermore, it is possible that some participants categorized as nonadherent in a given week did, in fact, meet the corresponding PA goal for that week; however, goal attainment could not be determined in this case if they did not submit their activity record on the website. Further, goal attainment relied on self-report of PA, which is well known for overestimation [49,50]. Providing technology in future studies to assist with objective PA measurement and self-monitoring could remove some of the barriers associated with manual posting of self-reported PA. Finally, a participant’s adherence status for each type of PA goal prescription was conditional on the preceding week’s level of adherence for that prescription and calculated independently from adherence to the other type of goal prescription; the influence of adherence at other time points or adherence to the other PA goal is an area for future study. Nevertheless, the approach selected allows for a preliminary examination of dynamic adherence to PA goals and self-monitoring.

### Conclusion

This study begins to identify key transition points in the behavior change process as it relates to PA adherence for step- and minute-based goals in a behavioral weight loss program and provides a useful framework for detecting responsiveness to program recommendations. Moreover, it may assist in shaping more effective PA recommendations in lifestyle programs, particularly during the early period of program initiation. The initial 2 months provide a window of opportunity to assist people in transitioning to adherent behavior, especially those who fail to meet the PA goals but engage in self-monitoring. As a result, researchers and clinicians should consider PA self-monitoring and goal attainment concurrently as an indication of an individual’s adherence status. Emphasis should be placed on strategies that help people start and reach the 2-month mark with consistent self-monitoring and attainment of PA recommendations, since this appears to be a formative period for continued adherence. Future research should investigate how these transitional patterns in PA adherence status are influenced by individual characteristics and contextual factors, as well as how they relate to weight loss and other health-related outcomes, to inform optimization of treatment recommendations.

## Appendix

Multimedia Appendix 1 CONSORT diagram.

Multimedia Appendix 2 Flow diagram summarizing transitions in weekly adherence status to (A) step-based goals and (B) minute-based goals by physical activity (PA) goal phase (N=212). Solid bars represent the marginal proportions of participants in each adherence category at a given time point; bands represent the proportion transitioning to another category or remaining in the same category at 1 time point, given the previous time point.

Multimedia Appendix 3 Flow diagram of transitions in weekly adherence status to (A) step-based goals and (B) minute-based goals across the graded physical activity (PA) goal phase (N=212). Solid bars represent the marginal proportions of participants in each adherence category at a given time point; bands represent the proportion transitioning to another category or remaining in the same category at 1 time point, given the previous time point.

## Abbreviations

BMI: body mass index
MVPA: moderate-to-vigorous physical activity
OR: odds ratio
PA: physical activity
RCT: randomized controlled trial
