# Supplementary figures and images for: Describing Transitions in Adherence to Physical Activity Self-monitoring and Goal Attainment in an Online Behavioral Weight Loss Program: Secondary Analysis of a Randomized Controlled Trial
Source: J Med Internet Res. 2022 Jan 28;24(1):e30673. doi: 10.2196/30673 (PMC8838543; doi:10.2196/30673)

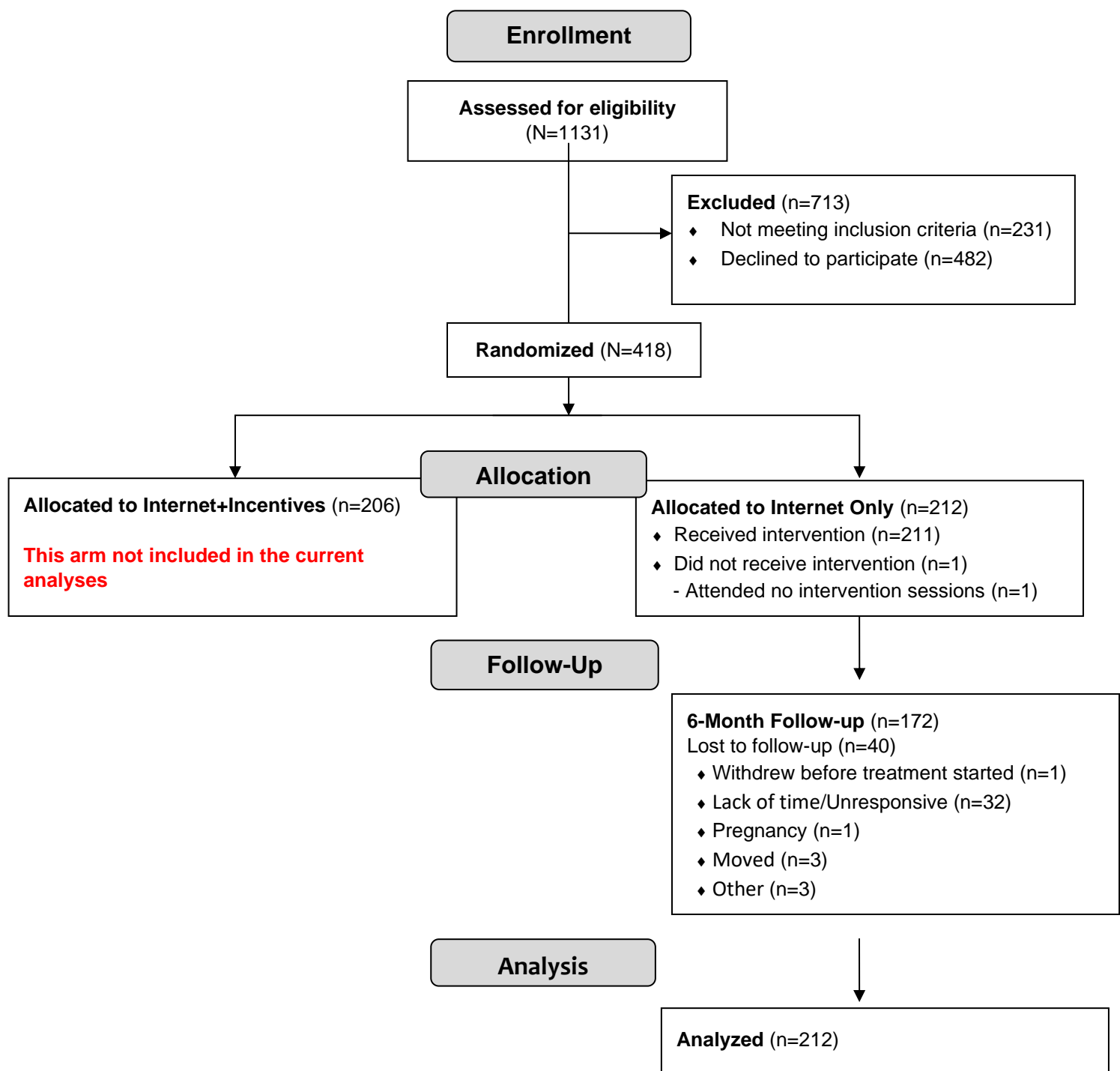

Supplement: Multimedia Appendix 1 [file jmir_v24i1e30673_app1.pdf]

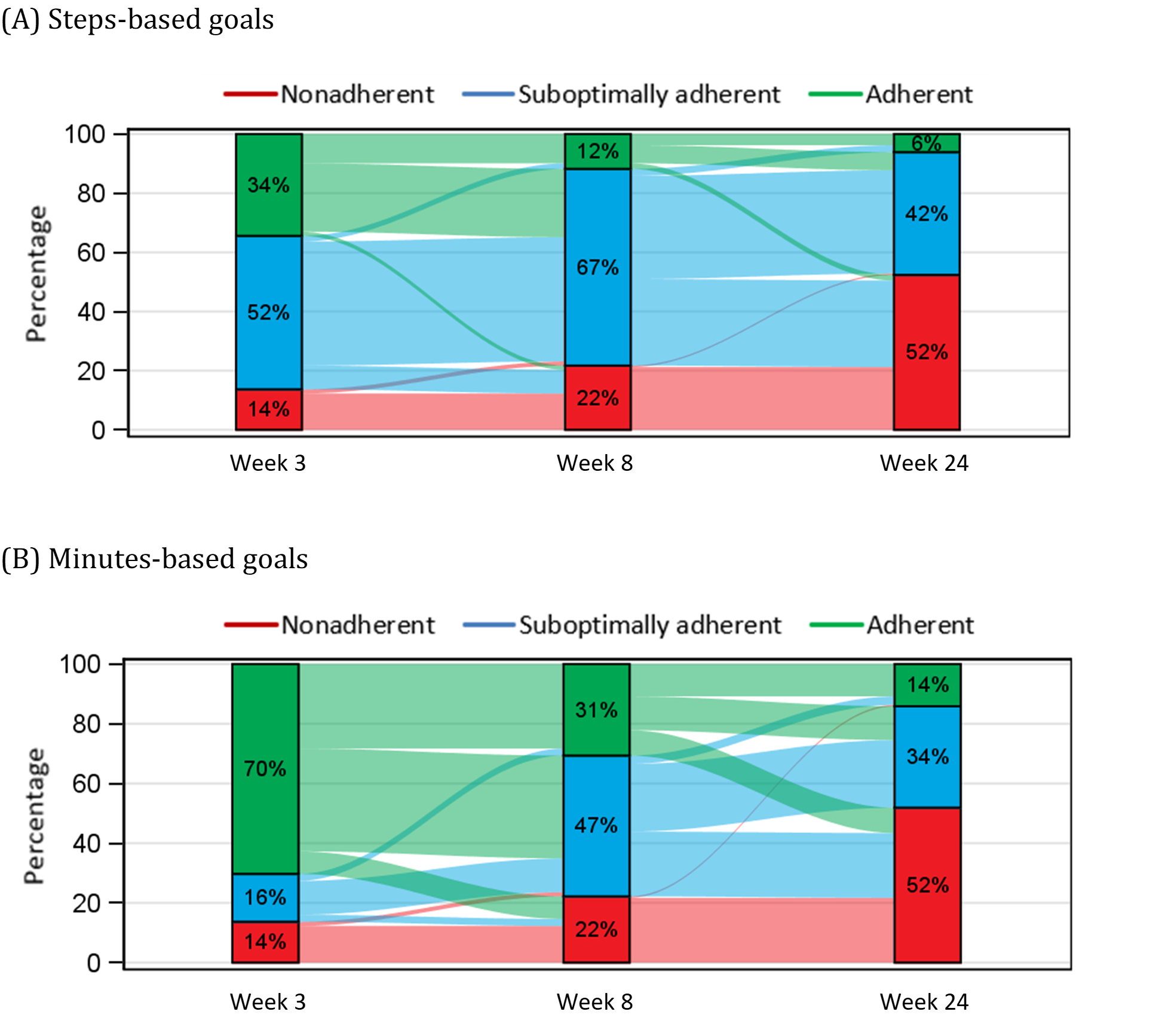

Supplement: Multimedia Appendix 2 [file jmir_v24i1e30673_app2.png]

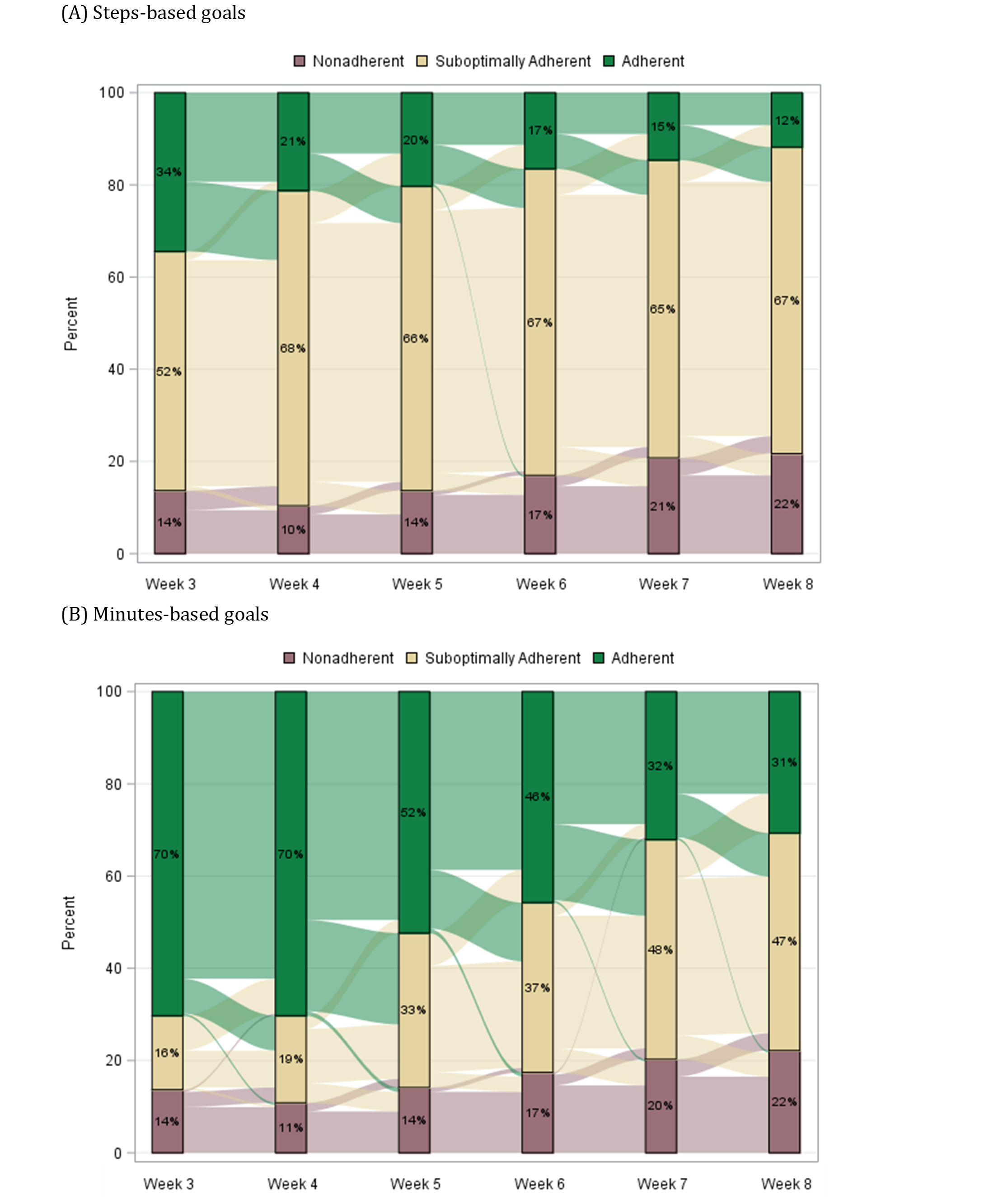

Supplement: Multimedia Appendix 3 [file jmir_v24i1e30673_app3.png]
